# Identification and Validation of a Five-Gene Diagnostic Signature for Preeclampsia

**DOI:** 10.3389/fgene.2022.910556

**Published:** 2022-06-14

**Authors:** Yu Liu, Xiumin Lu, Yuhong Zhang, Meimei Liu

**Affiliations:** Department of Obstetrics and Gynecology, The Second Affiliated Hospital of Harbin Medical University, Harbin, China

**Keywords:** preeclampsia, signature, expression profiles, bioinformatics, diagnosis

## Abstract

Preeclampsia is the leading cause of morbidity and mortality for mothers and newborns worldwide. Despite extensive efforts made to understand the underlying pathology of preeclampsia, there is still no clinically useful effective tool for the early diagnosis of preeclampsia. In this study, we conducted a retrospectively multicenter discover-validation study to develop and validate a novel biomarker for preeclampsia diagnosis. We identified 38 differentially expressed genes (DEGs) involved in preeclampsia in a case-control study by analyzing expression profiles in the discovery cohort. We developed a 5-mRNA signature (termed PE5-signature) to diagnose preeclampsia from 38 DEGs using recursive feature elimination with a random forest supervised classification algorithm, including *ENG*, KRT80, CEBPA, RDH13 and WASH9P. The PE5-signature showed high accuracy in discriminating preeclampsia from controls with a receiver operating characteristic area under the curve value (AUC) of 0.971, a sensitivity of 0.842 and a specificity of 0.950. The PE5-signature was then validated in an independent case-control study and achieved a reliable and robust predictive performance with an AUC of 0.929, a sensitivity of 0.696, and a specificity of 0.946. In summary, we have developed and validated a five-mRNA biomarker panel as a risk assessment tool to assist in the detection of preeclampsia. This gene panel has potential clinical value for early preeclampsia diagnosis and may help us better understand the precise mechanisms involved.

## Introduction

Preeclampsia is a common pregnancy complication and accounts for 2–8% of pregnancies worldwide (Ives et al., 2020). Preeclampsia dramatically increases the risk of all-cause mortality and has become a significant driver of maternal and perinatal morbidity and mortality, with over 70,000 maternal deaths and 500,000 fetal deaths worldwide every year (Armaly et al., 2018; Rana et al., 2019). Preeclampsia is not only associated with a long-term increased risk of cardiovascular and kidney disease but also contributes to neonatal respiratory distress syndrome and bronchopulmonary dysplasia (Phipps et al., 2019; Rana et al., 2019). Therefore, there is an urgent need to identify biomarkers for the early detection of preeclampsia to improve the treatment and outcomes of this prevalent disease.

Increasing evidence has suggested that preeclampsia is a multi-system and progressive disorder of pregnancy that can be characterized by new-onset hypertension and proteinuria (Grill et al., 2009). Abnormal placentation and the development of the maternal syndrome have been considered two stages implicated in the pathogenesis of preeclampsia (Roberts and Hubel, 2009; Staff, 2019). Many risk factors have been revealed and investigated during the past years, including a family history of preeclampsia, nulliparity, multiple pregnancy, advanced maternal age, *in vitro* fertilization, maternal comorbidities, maternal smoking, and so on (Kenny et al., 2015; Bartsch et al., 2016; Fox et al., 2019; Phipps et al., 2019). However, no helpful screening approach or marker was implemented in clinical practice. Although traditional risk-factor screening approaches, such as uterine artery doppler, serum biomarkers (such as Vitamin D Levels, VEGF, PlGF, sFLT-1, and sFLT1/PlGF ratio, soluble endoglin (sEng), pregnancy-associated plasma protein A (PAPP-A) and alpha fetoprotein (AFP), T-Lymphocytes, have been proposed for the prediction of preeclampsia (Mikat et al., 2012), there is much need to identify novel promising biomarkers for an early diagnosis prior to symptoms appearance.

In this study, we conducted a retrospectively multicenter discover-validation study and performed gene expression profiles analysis in a case-control study to identify potential biomarkers. Finally, we developed and validated an mRNA biomarker panel as a potential tool for the early diagnosis of preeclampsia.

## Materials and Methods

### Preeclampsia Cohorts Enrolled in This Study

Two independent preeclampsia cohorts were retrospectively collected from the Gene Expression Omnibus (GEO, https://www.ncbi.nlm.nih.gov/geo/), including 19 preeclamptic and 40 control placentas from Guo’s study (accession number GSE35574) (Guo et al., 2013), and 23 preeclamptic and 37 control placentas from Tsai’ study (accession number GSE25906) (Tsai et al., 2011).

### Gene Expression Data Processing and Analysis

Processed gene expression data profiled by Illumina human-6 v2.0 expression beadchip were obtained from GEO. All probes were mapped on the human reference genome (GRCh38), and finally, expression profiles of 18,355 protein-coding genes were obtained for further analysis. Differentially gene expression analysis was performed using the R package “limma” (version: 3.46.0). Those protein-coding genes were considered significantly differentially expressed with the cutoff value of |log2(fold change) | > 0.58 and false discovery rate (FDR) adjusted *p*-value < 0.1. Hierarchical clustering was performed with R software using the metric of canberra distance and ward. D clustering method. Functional enrichment analysis was conducted using Metascape (https://metascape.org) (Zhou et al., 2019).

### Identification of mRNA Signature for Preeclampsia Diagnosis

Biomarker selection is performed *via* the recursive feature elimination (RFE) with a random forest supervised classification algorithm with a 5-fold cross-validation analytic strategy used for biomarker discovery. An optimal gene panel was identified when the best compromise was reached between classification performance and smallness of the subset using the RFE method by recursively deleting the differentially expressed genes that are the less important (Zhou et al., 2018; Tronik-Le Roux et al., 2020). A diagnostic model was developed using the Least Absolute Shrinkage and Selection Operator (LASSO) as follows:

### Statistical Analysis

$$Diagnosis\;score=\frac{e^{w^{T}*x+b}}{1+e^{w^{T}*x+b}}$$

Statistical analyses were performed using R version 3.6.3. Precision-Recall curve, Kolmogorov-Smirnov plot and receiver operating characteristic (ROC) curves were used to evaluate the performance of the diagnostic model, and the area under the curve (AUC) values were calculated. The confusion matrix is also set up and the chi-square test was used to confirm the statistical significance for categorical variables.

## Results

### Study Design

We conducted a retrospectively multicenter discover-validation study to develop and validate a novel biomarker for preeclampsia diagnosis. In the discovery phase, we used the Guo cohort as a discovery cohort for identifying biomarkers and the Tsai cohort as an independent validation cohort for investigating the performance of biomarkers.

### Identification of Preeclampsia-Associated Genes

**FIGURE 1:**
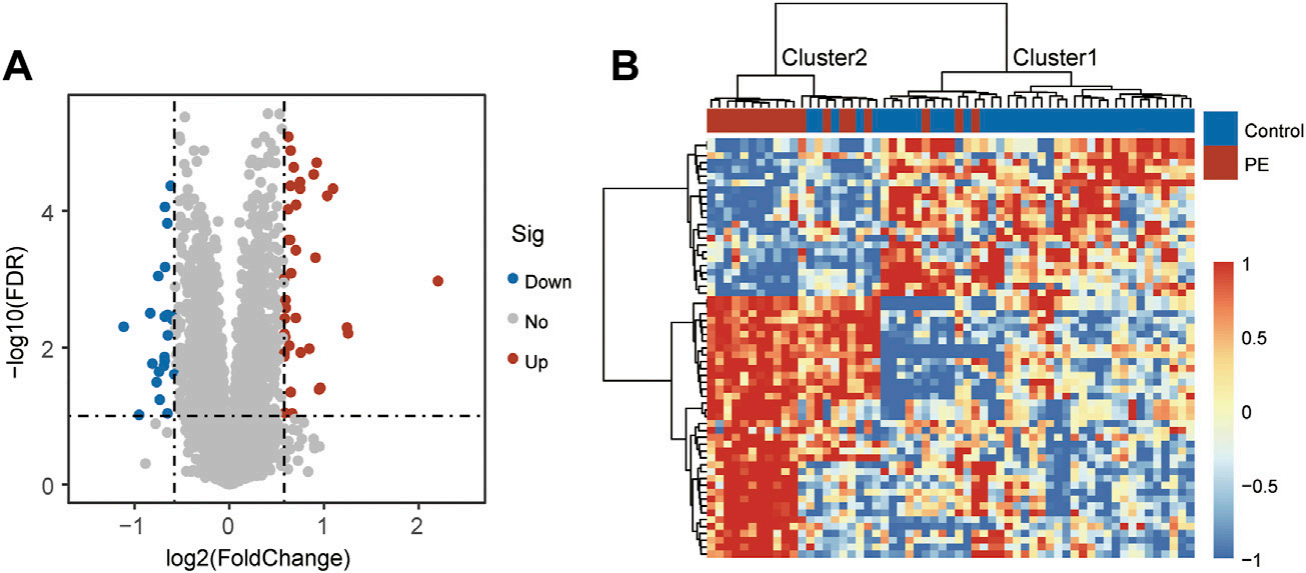
Identification of candidate mRNA biomarkers. **(A)** Volcano plot of differential gene expression. **(B)** Heatmap of unsupervised hierarchical clustering of differentially expressed genes between preeclamptic placentas and control placentas.

To identify preeclampsia-associated genes, we compared gene expression profiles between 19 preeclamptic and 40 control placentas from the discovery cohort and identified 61 significantly differentially expressed genes (DEGs) with |log2(fold change) | > 0.58 and FDR adjusted *p*-value < 0.1. Among them, 38 DEGs were observed to be significantly up-regulated, and 23 were down-regulated in preeclamptic placentas compared with control placentas (Figure 1A and Supplementary File S1). We performed unsupervised hierarchical clustering for samples in the discovery cohort based on the expression values of differentially expressed genes. The results separated preeclamptic and control placentas (Figure 1B). As shown in Figure 1B, most of the preeclamptic placentas (84.2%) were subdivided into Cluster 2, whereas most of the control placentas (87.5%) were revealed in Cluster 1 (Chi-square test *p* = 3.686e-07).

### Functional Characterization of Preeclampsia-Associated Genes

**FIGURE 2:**
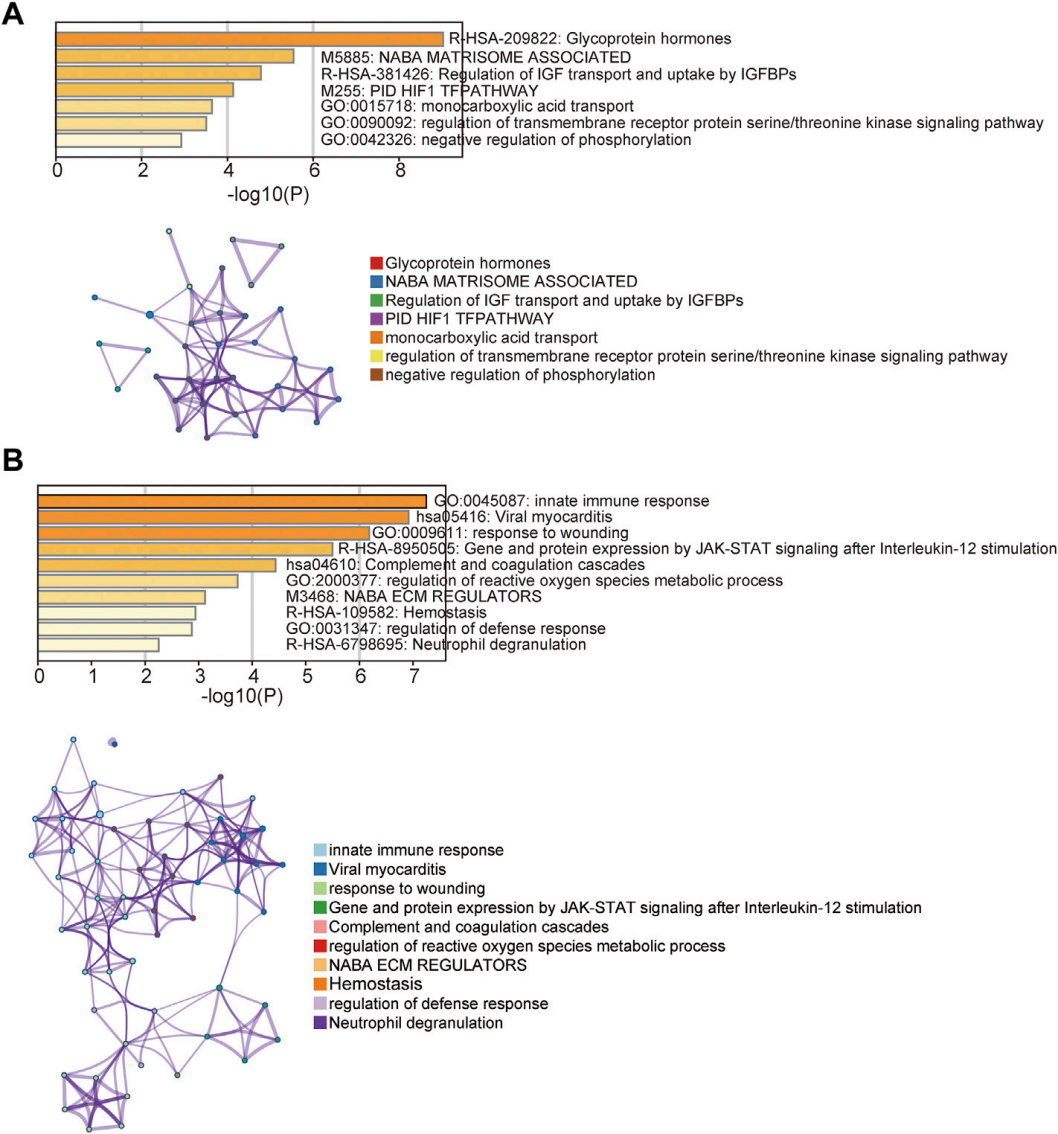
Functional enrichment analysis. **(A)** Enriched function terms and network of up-regulated differentially expressed genes. **(B)** Enriched function terms and network of down-regulated differentially expressed genes.

To explore the functional roles of these DEGs, we performed functional enrichment analysis for these 61 DEGs using the Metascape. We found that 38 up-regulated DEGs were significantly enriched in Glycoprotein hormones, NABA MATRISOME ASSOCIATED, Regulation of IGF transport and uptake by IGFBPs, PID HIF1 TFPATHWAY, monocarboxylic acid transport, regulation of transmembrane receptor protein serine/threonine kinase signaling pathway and negative regulation of phosphorylation (Figure 2A). Enrichment analysis of 23 down-regulated DEGs indicated that innate immune response, Viral myocarditis, response to wounding, Gene and protein expression by JAK-STAT signaling after Interleukin-12 stimulation, Complement and coagulation cascades, regulation of reactive oxygen species metabolic process, NABA ECM REGULATORS, Hemostasis, regulation of defense response and Neutrophil degranulation were involved (Figure 2B).

### Identification of Potential Biomarkers for Distinguishing Preeclamptic and Control Placentas

**FIGURE 3:**
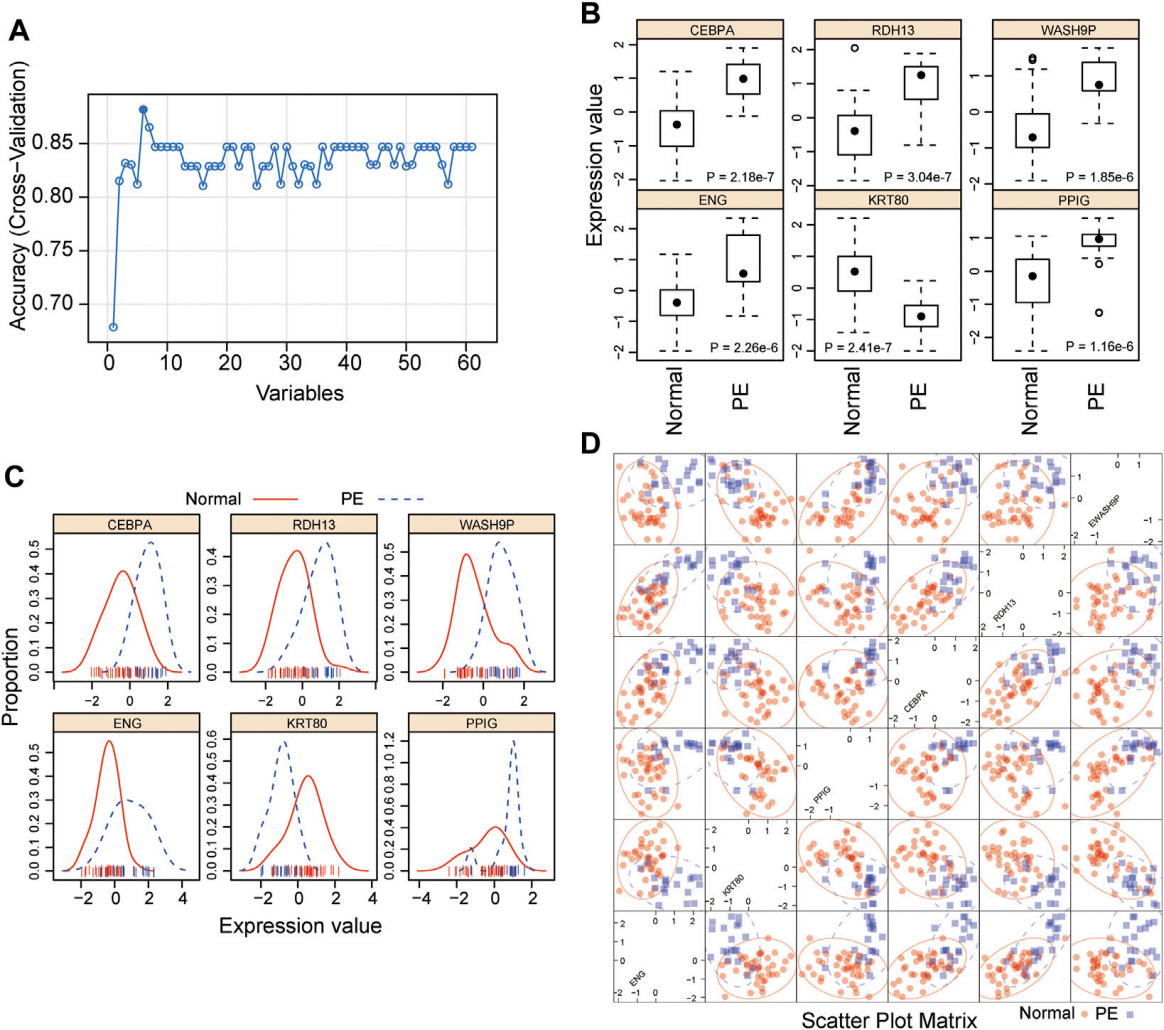
Development of a 5-mRNA gene panel for early preeclampsia diagnosis. **(A)** Boxplot showing the predicted accuracy of each combination constructed by a specific number of mRNA biomarkers. **(B)** Boxplot showing expression levels of six candidate mRNA biomarkers. **(C)** Distribution of expression levels of six candidate mRNA biomarkers. **(D)** Scatter Plot showing expression levels of six candidate mRNA biomarkers.

To identify potential biomarkers for distinguishing preeclamptic and control placentas, we conducted a feature selection analytic strategy using the RFE with random forest supervised classification with 5-fold cross-validation for all 61 DEGs in the discovery cohort. The above-mentioned analysis revealed that the combination of six DEGs achieved the highest predictive accuracy rate (Figure 3A). Of them, five biomarkers were found to be up-regulated and one down-upregulated in preeclamptic compared with control placentas (Figure 3B). As shown in Figure 3C, these six potential biomarkers revealed different expression patterns that distinguished preeclamptic samples from control samples (Figures 3C,D).

### Establishment of an mRNA Biomarker Panel for the Diagnosis of Preeclampsia

**FIGURE 4:**
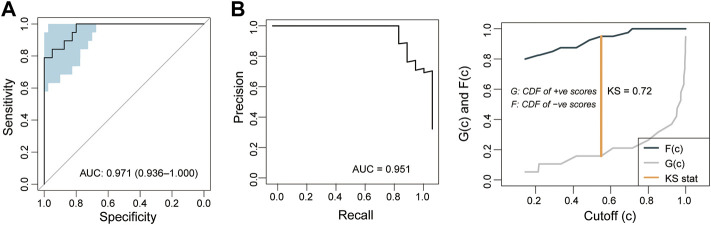
Performance evaluation of the 5-mRNA gene panel in the discovery cohort. **(A)** Receiver operating characteristics (ROC) curves for the 5-mRNA gene panel for preeclampsia diagnosis. **(B)** Precision-recall curves for the 5-mRNA gene panel for preeclampsia diagnosis.

The selected potential biomarkers were analyzed using the LASSO method to shrink the number of variables. Finally, five mRNA biomarkers (*ENG*, KRT80, CEBPA, RDH13 and WASH9P) were selected and used to construct a diagnostic model using the Logistic regression model (termed PE5-signature) (Table 1). We then calculated risk scores for each sample in the discovery based on the PE5-signature. ROC analysis revealed that the PE5-signature distinguished preeclampsia from controls with an AUC of 0.971 (95% CI 0.936-1.000), PRAUC of 0.971, a sensitivity of 0.842, a specificity of 0.950 (Figure 4A). The diagnostic accuracy was 91.5% with an F1 score of 0.865 (Figure 4B).

**TABLE 1:** Detailed information of five biomarkers in the signature.

Ensembl ID	HGNC symbol	Gene synonyms	Location	Expression	Weight
ENSG00000106991	ENG	CD105,END,HHT1,ORW, ORW1	Chr9: 130,577,291-130,617,035 (-)	Up	1.6615
ENSG00000167767	KRT80	KB20	Chr12: 52,562,780-52,585,784 (-)	Down	−0.9499
ENSG00000245848	CEBPA	C/EBP-alpha,CEBP	Chr 19: 33,790,840-33,793,470 (-)	Up	0.9640
ENSG00000160439	RDH13	SDR7C3	Chr 19: 55,550,476-55,582,659 (-)	Up	0.5016
ENSG00000279457	WASH9P	NA	Chr 1: 185,217-195,411 (-)	Up	1.3907

### Independent Verification of the PE5-Signature

**FIGURE 5:**
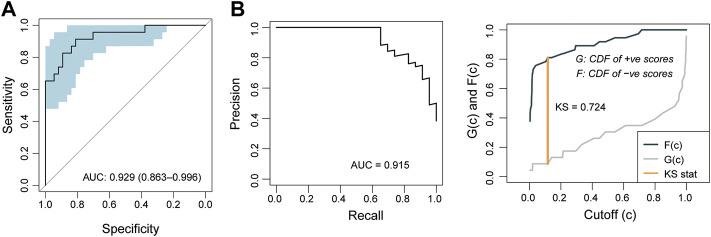
Independent validation of the 5-mRNA gene panel in the validation cohort. **(A)** Receiver operating characteristics (ROC) curves for the 5-mRNA gene panel for preeclampsia diagnosis. **(B)** Precision-recall curves for the 5-mRNA gene panel for preeclampsia diagnosis.

To further examine the robustness of the PE5-signature, we tested the predictive performance of the PE5-signature in another independent retrospective case-control cohort of 60 subjects from Tsai’s study. Similarly, the PE5-signature identified through the discovery cohort showed consistent predictive performance in the independent cohort. The PE5-signature could discriminate preeclampsia from controls with AUC of 0.929 (95% CI 0.863-0.996), PRAUC of 0.915, a sensitivity of 0.696, a specificity of 0.946 (Figure 5A). The diagnostic accuracy was 85% with a F1-score of 0.781 (Figure 5B).

## Discussion

Preeclampsia is the leading cause of morbidity and mortality for both mothers and newborns worldwide (Stocks, 2014). Despite extensive efforts made to understand the underlying pathology of preeclampsia, there is still no clinically useful effective tool for early diagnosis of preeclampsia, partly because of nonspecific clinical signs. Several studies have shown that dysregulated gene expression has been implicated in preeclampsia development (Meng et al., 2012; Vennou et al., 2020), highlighting the potential of gene expression as a promising biomarker for the early diagnosis of preeclampsia.

In this study, we conducted a retrospectively multicenter discover-validation study to develop and validate a novel biomarker for preeclampsia diagnosis. We identified 38 differentially expressed genes involved in preeclampsia in a case-control study by analyzing expression profiles downloaded from Guo’ study. Potential involvement of these differentially expressed genes in Glycoprotein hormones, monocarboxylic acid transport, regulation of transmembrane receptor protein serine/threonine kinase signaling pathway and negative regulation of phosphorylation, innate immune response, Viral myocarditis, response to wounding, Gene and protein expression by JAK-STAT signaling after Interleukin-12 stimulation, Complement and coagulation cascades, regulation of reactive oxygen species metabolic process, hemostasis, regulation of defense response and Neutrophil degranulation. Among 38 DEGs, five mRNA were finally selected for model construction through the RFE with random forest supervised classification with 5-fold cross-validation and LASSO method. These mRNA biomarkers consisted of the up-regulated *ENG*, CEBPA, RDH13 and WASH9P, and the down-regulated KRT80. *ENG* is a trans-membrane glycoprotein and its dysregulated expression may contribute to preeclampsia (Chang et al., 2011; Bell et al., 2013). *RDH13* encodes a mitochondrial short-chain dehydrogenase/reductase and its association with preeclampsia has been identified in several previous studies (Tsai et al., 2011; Trifonova et al., 2014). A large number of studies have provided ample evidence demonstrating that a combination of multiple genes manifests more efficiently than a single mRNA as a diagnostic tool (Liu et al., 2019; Sun et al., 2020; Bao et al., 2021; Zhao et al., 2021). Therefore, we developed a diagnostic signature using the logistic regression model to distinguish preeclampsia from controls with high diagnostic performance. This model also was further validated in another independent cohort, highlighting enormous potential applications for diagnosing preeclampsia.

However, several limitations should be noted in our study. First, the two cohorts both have a smaller sample size. Therefore, a larger validation cohort was needed to validate this signature’s reliability. Second, the cross-platform compatibility of this signature is not evaluated due to the limitation in available public data sets.

In conclusion, we have developed and validated a five-mRNA biomarker panel as a risk assessment tool to assist in the detection of preeclampsia. This gene panel has potential clinical value for early preeclampsia diagnosis and may help us better understand the precise mechanisms involved.

.

## Data Availability

Publicly available datasets were analyzed in this study. This data can be found here: The datasets used during the present study are available from Gene Expression Omnibus (www.ncbi.nlm.nih.gov/geo/query/acc.cgi?acc=GSE35574, and www.ncbi.nlm.nih.gov/geo/query/acc.cgi?acc=GSE25906).
